# Evaluation of the Neurocognitive Affective Model for the Prediction of Habitual 24‐Hour Physical Behavior

**DOI:** 10.1002/ejsc.70037

**Published:** 2025-08-12

**Authors:** Selina Schneider, Ulrich W. Ebner‐Priemer, Marco Giurgiu

**Affiliations:** ^1^ Institute of Sports and Sports Science Karlsruhe Institute of Technology (KIT) Karlsruhe Germany; ^2^ Department of Psychiatry and Psychotherapy Central Institute of Mental Health University of Heidelberg Medical Faculty Mannheim Mannheim Germany

**Keywords:** affective state, executive functions, physical behavior, wearables

## Abstract

Physical activity (PA) is recognized for its health benefits, including reduced risks of noncommunicable diseases. Despite recommendations for PA, global inactivity rates remain high. The neurocognitive‐affective model proposes that executive functions and affective responses to PA may influence habitual PA behavior. This cross‐sectional study aimed to test the model's pathways, hypothesizing that (A) executive functions are associated with PA‐induced affective responses, (B) affective responses are associated with habitual PA, and (C) the association between executive functions and PA. This study included 222 healthy university employees with predominantly sedentary occupations. Participants completed cognitive tasks (i.e., task switching, Stroop test, and numerical updating task) under laboratory conditions and an incremental treadmill test to assess PA‐induced affective responses. PA was tracked for at least 15 days using a multisensor system (thigh‐worn Move 4 and wrist‐worn Fitbit Inspire 2). Results showed that cognitive flexibility, as measured by task‐switching costs; working memory, as measured by percentage score of correct answers; and inhibitory control, as measured by difference of reaction time in congruent and incongruent trials, were not significantly associated with postexercise affect. Affective responses before and during exercise were positively associated with habitual moderate‐to‐vigorous PA and light PA, whereas task‐switching performance was inversely related to habitual MVPA. Exploratory analyses revealed significant correlations between affective responses during exercise and sleep duration, as well as between task‐switching performance and sleep duration. This study provides partial support for the neurocognitive‐affective model of PA. Future research should explore these pathways at different temporal resolutions and consider within‐person analyses.

## Highlights

- This study is one of the first empirical studies that tested the pathways of the neurocognitive affect‐related model in a sample of healthy adults.
- Executive function was not associated with postexercise affective responses.
- Affective responses before and during exercise were predictors of habitual PA. A higher Feeling Scale score at the midpoint of exercise was positively associated with habitual MVPA minutes. Similarly, participants who felt better before exercise also engaged in more habitual MVPA and LPA, emphasizing the importance of affect in promoting habitual PA.
- In exploratory analyses, this study extended the neurocognitive‐affective model to include sedentary behavior and sleep. It was found that better feelings before exercise were associated with reduced habitual sleep duration. Additionally, higher task‐switching costs were linked to longer habitual sleep duration. These findings suggest that both cognitive function and affective states may play roles in influencing overall 24‐h movement behavior, including nonexercise activities.

## 1 Introduction

Regular physical activity (PA) is associated with a reduced prevalence of noncommunicable diseases and mental illness (Warburton et al. 2007). For instance, chronic diseases such as Type 2 diabetes, cardiovascular diseases, and certain types of cancer have been linked to a lower PA (World Health Organization 2020). Most health benefits are observed when individuals transition from a sedentary lifestyle to regular PA with higher intensity levels, such as moderate‐to‐vigorous PA (MVPA) (Warburton and Bredin 2017). To achieve these effects, the World Health Organization (WHO) recommends that adults should engage in 150–300 min of moderate aerobic activity or 75–150 min of vigorous PA per week (World Health Organization 2020). However, despite multifaceted efforts to increase PA, the latest data indicate that the global age‐standardized prevalence of physical inactivity (i.e., not meeting PA recommendations) increased from 23.4% in 2000 and 26.4% in 2010 to 31.3% in 2022 (Strain et al. 2024).

Numerous theories aim to explain the determinants of PA behavior. Most of these theories fall within the frameworks of either social‐cognitive models (e.g., the theory of planned behavior) (Ajzen 1991) or humanistic/organismic models (e.g., self‐determination theory) (Rhodes et al. 2019). These frameworks are crucial for explaining why some individuals are active, whereas others are not. However, by focusing exclusively on cognitive aspects, these models only explain a modest portion of the variance (Sheeran et al. 2020; Szczuka et al. 2021). More recently, new models, such as the Wants and Aversions for Neuromuscular Tasks model (Stults‐Kolehmainen et al. 2023), the dual‐mode theory (Ekkekakis et al. 2011), the upward spiral theory of lifestyle change (Van Cappellen et al. 2018), the integrative framework (Williams et al. 2019), and the affective‐reflective theory (Brand and Ekkekakis 2018), have emerged. Additionally, in the context of PA, the multiprocess action control approach (Rhodes et al. 2021) and the PA adoption and maintenance model (Strobach et al. 2020) take the dual‐process approach into account. These models build on hedonic theories of behavior and acknowledge that affective (often referred to as Type 1) processes play a significant role in shaping human health and PA behaviors. Type 1 processes, which are more automatic and emotional, contrast with rational, reflective (Type 2) processes (Kahneman 2011). A systematic review (Rhodes and Kates 2015) provides comprehensive evidence on the predictive value of affective responses during exercise. The review synthesizes findings from numerous studies and concludes that positive affective experiences during exercise are consistently associated with higher levels of future PA. The authors argue that affective responses may influence motivational processes and the formation of exercise habits, thereby serving as a key mechanism in sustaining PA over time.

**FIGURE 1:**
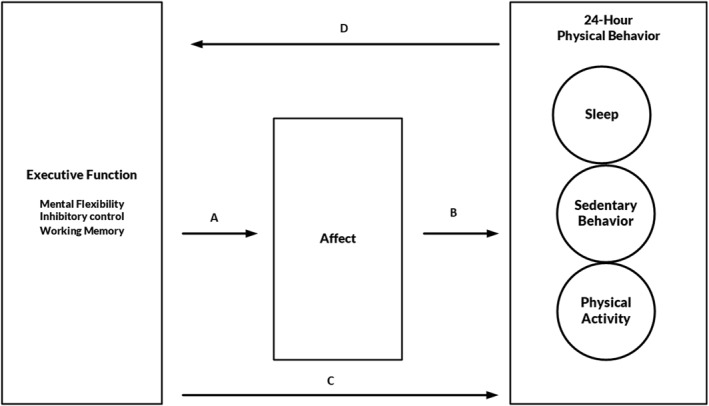
A modified version of the neurocognitive affect‐related model for the prediction of upcoming 24‐h physical behavior.

An example of a dual‐process model is the neurocognitive‐affective model (Edwards et al. 2017). The model postulates an interaction between executive functions, affective states, and habitual PA behavior. Specifically, this model includes three pathways (see also Figure 1): (i) Neurocognition, particularly cognition based on executive function, may significantly influence exercise‐induced affective responses (Pathway A); (ii) an individual's affective response from exercise may predict habitual exercise behavior (Pathway B); and (iii) a bidirectional relationship where executive function indirectly influences habitual exercise behavior through affective responses to exercise, whereas exercise itself plays a crucial role in enhancing executive functioning (Pathways C and D) (Edwards et al. 2017).

Empirical evidence supports the pathways between these constructs, showing that regular participation in PA leads to structural and functional changes in the brain. This occurs through repeated training sessions, where cellular processes are enhanced by increased concentrations of neurotrophic factors and growth factors (El‐Sayes et al. 2019). These changes in the brain, in turn, may facilitate future engagement in PA and thus indicate a bidirectional association (Loprinzi et al. 2013). Central cognitive processes that are positively influenced by PA are executive functions, which are controlled by the prefrontal cortex and are responsible for executing goal‐directed behaviors and inhibiting goal‐inconsistent behaviors (Esposito et al. 2016). According to Diamond's study, executive functions enable mentally playing with ideas, taking time to think before acting, handling novel and unexpected challenges, resisting temptations, and maintaining focus. The core executive functions include inhibition control (e.g., impulsive actions and interference control), working memory, and cognitive flexibility (e.g., viewing situations from different perspectives) (Diamond 2013).

Executive functions have been shown to moderate the relationship between intention and behavior across various behavioral domains, including PA (Hall et al. 2008). Various studies demonstrate the role of executive functions as a moderator of the intention–behavior relationship, both in PA (Pfeffer and Strobach 2017) and other health behaviors (Allan et al. 2011). Further studies have shown how executive functions can support the self‐regulation of PA behavior. Pfeffer and Strobach (2020) focused on how planning intervention impacts PA in the short term. In a randomized controlled trial with 200 students, the planning group showed a significant increase in PA compared to the control group. Planning improved PA, especially for those with high intentions and low executive function updating, indicating that forming plans can help compensate for cognitive limitations and support behavior change (Pfeffer and Strobach 2020). A study by Forestier et al. (2023) found that trait self‐control and self‐control resources significantly predicted healthier behaviors, such as increased PA. Among executive functions, cognitive flexibility (switching) was indirectly linked to health behaviors via self‐control resources (Forestier et al. 2023).

In addition to directly regulating behavior through executive control processes, executive functions may also indirectly influence PA through the modulation of affective responses. An affective response (e.g., experiencing feelings of pleasantness or unpleasantness), which impacts habitual PA behavior, can be shaped by the cognitive interpretation of an individual's past physiological experiences (Rhodes and Kates 2015). It is suggested that affective responses related to PA can even occur independently of executive‐controlled processes (Rhodes and Kates 2015).

The evaluation of the neurocognitive affect‐related model using empirical data has so far been limited to a single study with a small sample size (*N* = 32). In particular, Loprinzi and colleagues employed a 1‐week prospective, multisite design study (Loprinzi et al. 2020). Participants completed a bout of treadmill exercise, with affect and arousal assessments before, during, and after the exercise session. Objective measures of executive function were captured during this laboratory visit. Thereafter, habitual engagement in PA was captured in a 7‐day accelerometry measurement period. The authors observed some associations between executive function and light‐intensity PA (LPA) in the future, as well as associations between executive functions and postexercise affect. Overall, the first empirical evaluation indicated partial support for a cognitive‐affective model of PA (Loprinzi et al. 2020).

Based on the study of Loprinzi et al. (2020), this study aims to evaluate the pathways of the neurocognitive‐affective model in a larger sample size among healthy working adults. Additionally, this study is one of the very few that combines field research with laboratory research. This combination strengthens this study by ensuring both high ecological validity and reliable assessment under controlled laboratory conditions. In line with the conceptual model by Edwards et al. (2017), we hypothesize (A) executive functions are significantly associated with PA‐induced affective responses; (B) PA‐induced affective response is significantly associated with habitual PA; and (C) executive functions are significantly associated with habitual PA. Additionally, we conducted exploratory analyses to test whether affective responses might mediate the effects of executive functions on habitual PA.

Moreover, in line with the growing recognition of the 24‐h movement behavior paradigm within the PA research community (Rosenberger et al. 2019), our study adopts an exploratory approach that extends beyond PA to include sedentary behavior and sleep (see Figure 1). This integrated perspective acknowledges that time spent in one behavior inherently displaces time spent in another, underscoring the compositional and interactive nature of daily movement behaviors. Sedentary behavior and sleep are themselves associated with distinct physiological and psychological outcomes and are influenced by both affective and cognitive factors. For instance, sedentary time is often shaped by automatic behavioral patterns, environmental cues, and executive function capacities (e.g., self‐regulatory control), whereas sleep patterns have been linked to emotional states, stress regulation, and circadian processes. There is also empirical evidence that affective responses may modulate sleep quality and duration (Ong et al. 2017; Tavernier et al. 2016), suggesting shared regulatory pathways across movement behaviors. By situating PA, sedentary behavior, and sleep within a unified cognitive‐affective framework, this study aims to provide a more holistic understanding of how neurocognitive and affective processes collectively shape habitual 24‐h behavior patterns. As a secondary exploratory purpose, we tested whether the associations between PA, affective responses, and executive functions were influenced by the measurement of PA (i.e., research‐grade vs. commercial device).

## 2 Materials and Methods

### 2.1 Participants

This study is a secondary analysis of a within‐person encouragement design. Between October 2022 and December 2023, a total of 222 university employees were recruited for this study. The inclusion criteria required participants who spent, based on self‐reported perception, the majority of their working day in a sedentary position (e.g., office workers or those in similar occupations) and to be capable of performing daily life activities, indicating they had no current physical injuries or mental illnesses. This study was approved by the Ethics Committee of the Karlsruhe Institute of Technology (KIT). All participants received comprehensive written and oral information about the study procedures before providing their written informed consent. Participation was voluntary, and participants had the option to withdraw at any point. Participants received a fitness tracker worth €100 as an incentive to take part (for details, see Giurgiu et al. 2024).

### 2.2 Study Procedures

After recruiting via flyers, mailing, and word of mouth, participants completed an initial in‐person session of approximately 3 h under laboratory conditions. During this appointment, participants completed a set of cognitive tasks, filled in questionnaires, and performed an incremental treadmill test to volitional exhaustion with a 6‐2‐1 protocol (i.e., starting with 6 km/h and changing the speed by 1 km/h after every 2 min). After the in‐person session, participants started the ambulatory assessment phase for a minimum of 15 working days. The weekend days were scheduled as break days without assessments to maintain motivation and thus compliance.

### 2.3 Measures

#### 2.3.1 Executive Functions

Participants performed three different cognition tasks on a stationary computer. First, based on the task of Rogers and Monsell (1995), participants completed the task switching test to measure cognitive flexibility (Rogers and Monsell 1995). In particular, participants were presented with a 2 × 2 matrix on the screen with different pairs of letters and numbers. In the upper two fields, the participants focused only on the letter and decided as fast as possible whether the letter was a consonant (G, K, M, R) or a vowel (A, E, I, U). In the lower two fields, the participants focused only on the number and decided as fast as possible whether the number was even (2, 4, 6, 8) or odd (3, 5, 7, 9). To enter the result, the participants either pressed the Y key on the keyboard for consonant letters or odd numbers or pressed the M key for vowel letters or even numbers. The test comprised four blocks with 48 trials each. At the start of the test, the four rectangles with the first letter‐number pair appeared in the top right‐hand square. A pair was displayed until the participant gave their answer or until 5000 ms had elapsed. This was followed by a 150‐ms interval before the next letter‐number pair was displayed in the following square (clockwise). If the participant's answer was incorrect or the time of 5000 ms was reached, the word “error” appeared for 500 ms before the next pair was displayed. After each of the four individual blocks of 48 trials, the percentage of correct answers appeared, as well as a break, which could be ended independently by the test person by pressing the “Y” or “M” key, and thus the next block could be started. The main parameter used in statistical analyses was the switch costs (i.e., difference in reaction time between switch trials and nonswitch trials), with lower switch costs reflecting a smaller difference between the different trial types, thereby indicating better performance (Gura‐Solomon et al. 2024). This parameter is expected to be negatively associated with affect and PA. Further, we descriptively reported the percentage of all correct answers in switch trials (trials with task switching), the percentage of all correct answers in nonswitch trials (trials without task switching), and the average reaction time of correct switch trials and nonswitch trials (Dick et al. 2024).

Second, the participant completed the Stroop task as an indicator of inhibition control. In line with the previously reported task procedures (Stroop 1935), the task consisted of 84 trials in which one of four color words in capital letters (RED, GREEN, BLUE, or YELLOW) was presented on the screen in random order in different font colors (red, green, blue, or yellow). In congruent conditions, the font color and the meaning of the word matched (e.g., RED written in red font color). In the case of incongruent conditions, the font color differed from the meaning of the word (e.g., RED written in blue font color). The test included 28 congruent (seven congruent trials per color) and 28 incongruent (seven incongruent trials per color) trials in randomized order. In addition, 28 randomized controlled trials were integrated, with a triangle appearing instead of a word in one of the four colors (seven times per color). Participants were asked to indicate the font color (or the color of the triangle) on each trial by pressing the appropriate arrow key on a keyboard. Red corresponded to the up arrow, yellow to the right arrow, green to the down arrow, and blue to the left arrow. The four keys were each covered with a matching‐colored dot to make it easier for the participants to assign them. After the participant responded, or after 8000 ms, there was an interstimulus interval of 500 ms before the next word appeared on the screen. After completing the 84 trials, the test was terminated. The main parameter used in statistical analyses was the difference between the reaction time of all congruent trials and all incongruent trials (Xu et al. 2024). A smaller difference between the different trial types indicates better performance. This parameter is expected to be negatively associated with affect and PA. Further, we descriptively reported the reaction time of all congruent trials and all incongruent trials, as well as the percentage of correct responses (Schultz et al. 2024; Descollonges et al. 2024; You et al. 2024).

Finally, as a third test, the participants completed the numerical updating task (Salthouse et al. 1991) as an indicator of working memory performance. The task was set up with four digits in a horizontal array, which had to be initially memorized and then updated according to arithmetic operations (i.e., addition and subtraction in the range of ± 1–8) that were presented sequentially in a random manner. The presentation time for the four starting digits was 4000 ms, followed by an interstimulus interval of 500 ms. In total, participants completed four sessions and 12 trials, resulting in a total of 48 trials. During each trial, eight updating operations had to be applied with varying presentation times of 6000 ms during the first session, 3000 ms during the second session, 1500 ms during the third session, and 750 ms during the fourth session. Participants had 4000 ms per digit to enter the result via tapping on a digit block with the mouse pad. For the analysis, the total score was used as the percentage value of all correct answers for the entire test (Salthouse et al. 1991). The higher the score, the better the cognitive performance. This parameter is expected to be positively associated with affect and PA. All tests were presented with the software Presentation (version 23.0, build 10.27.21; Neurobehavioral Systems Inc., https://www.neurobs.com/).

#### 2.3.2 Affect

Participants reported their current affective states in the laboratory using self‐reported scales before, during, and after a treadmill task while measuring respiratory gas via spiroergometry with the CORTEX system (MetaSoft Studio software, version 3.02.36). Participants completed an incremental treadmill test to volitional exhaustion. The task started at a speed of 6 km/h and was increased by 1 km/h every 2 min. The participants were able to end the test independently by straddling off the treadmill. Before, during, immediately after, and after 10 min of spiroergometry, the participants were asked to complete the affective state on scales. Because participants should not speak during spiroergometry due to possible distortion of the values, the scales were presented on posters. Participants indicated their answers by pointing to the corresponding number on the following scale: The *Feeling Scale* (FS) (Hardy and Rejeski 1989), which is widely used for assessing affective experiences during PA (Evmenenko and Teixeira 2022), was completed by the participants before, during, immediately after, and 10 min after the inpatient spiroergometry. The participants were asked to rate on a scale from minus five (very bad) to five (very good) how they generally felt at the time of the question. In this context, the validated German version was used (Thorenz et al. 2024). Next to the FS ratings before, immediately after, and after 10 min of spiroergometry, we used the rating of the FS that was collected at the midpoint during the treadmill task. During the activity, affect was assessed in the final seconds of each stage. The FS midpoint value was calculated, indicating the score the participant reported at the midpoint of their individually performed stages of the cardiopulmonary exercise test. For an odd number of stages, the midpoint stage was rounded up, and the score from that stage was used as the midpoint value (e.g., 7 stages performed; midpoint: 3.5; rounded up: 4). Before and 10 min after spiroergometry, the survey took place on a paper questionnaire or a tablet (software: movisensXS, version 3.0.4).

#### 2.3.3 Device‐Based Assessment of Physical Behavior

To capture daily physical behavior, participants were instructed to wear the Move 4 accelerometer (movisens GmbH, Karlsruhe, Germany, movisens.com) on their right thigh and the Fitbit Inspire 2 (Fitbit LLC, San Francisco, United States, fitbit.com) on their wrist continuously for 24 h per day (Monday to Friday). The *Move 4* accelerometer captured movement and nonmovement behaviors with a range of ± 16 g and a sampling frequency of 64 Hz. Raw acceleration data were stored on an internal memory card and were processed by a band‐pass filter (0.25–11 Hz) to eliminate artifacts. Previous validation studies have shown that the Move accelerometer is appropriate to differentiate between sitting/lying and upright body postures (Giurgiu et al. 2020) and to capture intensity levels of PA (Anastasopoulou et al. 2012). To parameterize the accelerometer raw signals, we calculated relevant parameters (i.e., body position and step count) in 1‐min intervals by using the software DataAnalyzer (version 1.16.8; movisens.com). We classified all awake minutes into the following categories: sedentary (i.e., sitting/lying body posture), light PA (i.e., upright body posture and < 100 step counts per minute), and MVPA (i.e., upright body posture and ≥ 100 step counts per minute). Sleep times were manually determined by visual inspection of acceleration signals, step counts, and temperature using the UnisensViewer application (version 1.16.46.0; movisens.com).

Participants were asked to wear the Fitbit Inspire 2 tracker on the wrist. Previous studies validated Fitbit trackers with mixed findings. For example, a systematic review indicates that the Fitbit Charge and Fitbit Charge HR were consistently shown to have good accuracy for step counts but failed to be accurate in measuring energy expenditure (Germini et al. 2022). Fitbit devices use proprietary algorithms to continuously measure biobehavioral features at up to a one‐second sampling rate (Bagot et al. 2018) with photoplethysmography and a triaxial accelerometer. To have access to the collected Fitbit data, the used Fitbit accounts were linked to the Fitabase platform (Fitabase, San Diego, United States, fitabase.com). In detail, the Fitbit data were continuously uploaded online to the cloud‐based platform. After data collection, the Fitbit data were manually downloaded from the Fitabase platform, which allows receiving a variety of PA parameters on different temporal resolutions, such as every minute to day‐level information. In the analyses, we used the intensity parameters (i.e., sedentary minutes, lightly active minutes, fairly active minutes, very active minutes) based on proprietary algorithms. We removed all wearable data that did not fulfill the following wear‐time criteria of at least 3 valid days (i.e., wear time ≥ 20 h per day).

### 2.4 Statistical Analyses

Prior to statistical analyses, preprocessing steps were conducted. The Fitbit data collected during the study period were downloaded as an Excel file with minute‐level data for all participants from Fitabase and further processed using Julia from Pluto.jl. Demographic characteristics of the participants were presented using descriptive statistics, with means, standard deviations, minimum, and maximum values calculated for age, body mass index (BMI), and sex distribution. To test the hypotheses, we conducted multiple regression analyses. Additionally, we conducted exploratory analyses to test for potential mediation effects. Statistical significance was set at a *p*‐value of less than 0.05. All analyses were performed using SPSS version 28 (IBM Corp., Armonk, NY, USA) in combination with the PROCESS macro (version 4.2; Hayes 2022).

## 3 Results

### 3.1 Descriptive Results

Several participants were excluded from the final analysis for various reasons: withdrew from the ambulatory assessment study (*N* = 3), had technical difficulties with the wearable (*N* = 1 (Move); *N* = 9 (Fitbit)), removed the sensor during the night (*N* = 17), and did not provide at least three valid days with ≥ 20 h wear time (*N* = 7 (Move); *N* = 14 (Fitbit)). Consequently, the final sample comprised 194 participants, 55.7% of whom were female, with an average age of 35.8 ± 10.6 years and a mean body mass index (BMI) of 24.04 ± 3.3 kg/m^2^ (see Table 1 for more details). In total, 194 participants provided valid data from the Move 4 sensor with an average of 11.1 (SD = 4.1) valid days, ranging from 3 to 21 days. Participants had worn the device on average for 22 h (SD = 0.5) per day, with a range from 20.8 to 23 h. Data from the Fitbit tracker were obtained from 179 participants, who provided mean valid data of 15.4 (SD = 6.9) days, ranging from 3 to 39 days. Participants had worn the tracker on average for 23.4 h (SD = 0.6) per day, with a range from 20.2 to 24 h. On average, time spent in the 24‐h movement behavior differed for each sensor type. In particular, the measurement differences between the Move and the Fitbit tracker were on average 4.7 min for sleep, 47.3 min for sedentary behavior, 91.6 min for LPA, and 34.8 min for MVPA, respectively. To compare the differences between both sensor types, we descriptively summarized the differences from the wake activities in a cross‐table (see Table 1). We focused only on minutes if both devices had been worn simultaneously (i.e., over 3.6 million minutes). From both devices, 1.620.197 min of sedentary behavior, 271.752 min in LPA, and 36.411 min in MVPA were simultaneously recorded. The activity recordings from both devices yielded an accuracy of 90.3% for sedentary behavior, 89.47% for LPA, and 96.59% for MVPA (see Table 2). However, when analyzing the sensitivity and specificity, we observed larger differences in activity minutes (i.e., MVPA (sensitivity 27%; specificity 99%); LPA (sensitivity 51%; specificity 96%); sedentary behavior (sensitivity 96%; specificity 85%)).

**TABLE 1:** Tracked minutes from the Move 4 sensor and Fitbit of wake activities (*N* = 194).

		Sedentary Fitbit (min)		LPA Fitbit (min)		MVPA Fitbit (min)	
		False	True	False	True	False	True
Sedentary Move (min)	False	1′639′876	66′333	1′412′045	294′164	1′615′479	90′730
	True	283′752	1′620′197	1′665′976	237′973	1′858′170	45′779
LPA Move (min)	False	1′597′557	1′620′908	2′958′080	260′385	3′136′275	82′190
	True	326′071	65′622	119′941	271′752	337′374	54′319
MVPA Move (min)	False	1′864′850	1′685′819	3′040′899	509′725	3′450′526	100′098
	True	58′823	711	37′122	22′412	23′123	36′411

**TABLE 2:** Participants' characteristics (*N* = 194).

	Mean ± SD	Min	Max
Age (years)	35.74 ± 10.61	20.18	64.76
BMI (kg/m^2^)	24.04 ± 3.30	17.87	35.25
Sex (% female)	55.7%		
VO_2max_, spiroergometry (mL/min/kg)^a^	39.87 ± 8.34	16	64

^a^ Data available from 191 participants.

The descriptive statistics for the sample and the physical behavior parameters are shown in Tables 2 and 3, respectively. The descriptive statistics for the cognitive parameters of working memory, cognitive flexibility, and inhibitory control are shown in Table 4. Affective parameters of the Feeling Scale before, during, and after PA are summarized in Table 4.

**TABLE 3:** Descriptive statistics for the 24‐h movement behavior parameters.

	Mean ± SD	Min	Max
Move 4 (*N* = 194)			
Sleep (min/day)	425.6 ± 59.3	271.3	635.2
Sedentary behavior (min/day)	726.5 ± 70	510.6	984.8
LPA (min/day)	146.8 ± 40.7	17.3	244.7
MVPA (min/day)	20.8 ± 12.1	0.7	64
Fitbit Inspire 2 (*N* = 179)			
Sleep (min/day)	430.2 ± 46.3	259	583.4
Sedentary behavior (min/day)	679.2 ± 63.7	471	878
LPA (min/day)	238.5 ± 53.9	102.2	404.4
MVPA (min/day)	55.6 ± 30	6.1	204

**TABLE 4:** Descriptive statistics for the cognitive and affective parameters (*N* = 194).

	Mittelwert ± SD	Min	Max
Working memory—numerical updating task			
Absolute score (%)	68.97 ± 12.16	5.73	95.83
Cognitive flexibility—task switching			
Absolute score switch trials (%)	93.76 ± 7.34	28.26	100
Absolute score nonswitch trials (%)	96.5 ± 6.31	31	100
Reaction time correct score switch trials (ms)	1484.92 ± 496.12	660.52	4144.11
Reaction time correct score nonswitch trials (ms)	1003.23 ± 367.51	535.78	3981.52
Switch cost (ms)	481.69 ± 289.52	−540.70	1868.73
Inhibition control—Stroop			
Reaction time congruent (ms)	744.9 ± 143.73	493	1211
Reaction time incongruent (ms)	860.93 ± 192.56	550	1651
Difference of reaction time congruent and incongruent (ms)	116.04 ± 97.23	−36	563
Absolute score (%)	98.18 ± 2.50	73.81	100
Feeling Scale (FS)			
Pre^a^	2.81 ± 1.48	−1	5
Midpoint^a^	2.14 ± 1.54	−2	5
Post^b^	0.14 ± 2.37	−5	5
Post 10 min^a^	2.72 ± 1.72	−4	5

^a^ Data available from 191 participants.

^b^ Data available from 189 participants.

As a descriptive measure, correlation analyses between sociodemographic variables and study variables revealed various significant correlations. In particular, sex was negatively correlated with FS pre (−0.145; *p* = 0.032) and MVPA of Fitbit (−0.277; *p* ≤ 0.001) and positively correlated with LPA of Fitbit (0.194; *p* = 0.009). Moreover, BMI was negatively correlated with working memory score (−0.164; *p* = 0.014) and LPA of Fitbit (0.194; *p* = 0.009), as well as positively correlated with switch cost (0.149; *p* = 0.027). Age was negatively correlated with working memory score (−0.334; *p* < 0.001), FS midpoint (−0.298; *p* < 0.001), sleep of Fitbit (−0.247; *p* < 0.001), and sleep of Move (−0.281; *p* < 0.001). Age was positively correlated with switch cost (0.168; *p* = 0.012), difference of reaction time of congruent and incongruent Stroop trials (0.238; *p* < 0.001), LPA of Fitbit (0.168; *p* = 0.025), and LPA of Move (0.169; *p* = 0.019).

### 3.2 Hypothesis A (Executive Functions → Affect)

To test Hypothesis A, we calculated the effects of the three main predictors of executive functions (absolute score (numerical updating task), switch cost (task switching), difference of reaction time congruent and incongruent (Stroop)) on affect (FS pre, FS midpoint, FS post, FS post 10) while controlling for sociodemographic predictors (sex, age, BMI), thus resulting in 12 different models (see Table 5). Based on the *F*‐tests, all models showed significant associations, thus indicating that a model as a whole contributed to the explanation, with a range from 3.3% (updating task—FS pre) to 9% (Stroop task—FS midpoint). However, none of the main predictors of executive functions were significantly associated with the affect outcomes. Only the covariates age and sex emerged as significant predictors within the FS midpoint model, indicating that older participants and male participants reported lower affect at the midpoint of the activity.

**TABLE 5:** Multiple regression models for Hypothesis A.

	Model 1 (FS pre)	Model 2 (FS midpoint)	Model 3 (FS post)	Model 4 (FS post 10)
(a) Absolute score (numerical updating task)	0.016 (0.009)	−0.001 (0.009)	−0.014 (0.015)	0.002 (0.011)
Sex	−0.373 (0.213)	−0.492 (0.214)*	−0.069 (0.351)	−0.134 (0.252)
Age	−0.012 (0.010)	−0.036 (0.011)**	−0.004 (0.017)	−0.022 (0.012)
BMI	0.045 (0.032)	−0.052 (0.033)	0.008 (0.053)	0.012 (0.038)
(b) Switch cost (task switching)	−0.000036 (0.000)	0.00005 (0.000)	0.001 (0.001)	0.000 (0.000)
Sex	−0.400 (0.215)	−0.489 (0.214)*	−0.062 (0.347)	−0.137 (0.251)
Age	−0.127 (0.010)	−0.035 (0.010)**	−0.006 (0.017)	−0.021 (0.012)
BMI	0.039 (0.033)	−0.052 (0.033)	0.004 (0.053)	0.013 (0.038)
(c) Difference of reaction time congruent and incongruent (Stroop)	0.000061 (0.001)	0.000 (0.001)	0.002 (0.002)	0.000 (0.001)
Sex	−0.401 (0.215)	−0.495 (0.214)*	−0.066 (0.351)	−0.133 (0.252)
Age	−0.018 (0.011)	−0.036 (0.011)**	−0.003 (0.017)	−0.022 (0.012)
BMI	0.039 (0.033)	−0.051 (0.032)	0.017 (0.053)	0.010 (0.038)

*Note: B* (SE), unstandardized estimates and standard errors.

* *p* < 0.05.

** *p* < 0.001.

### 3.3 Hypothesis B (Affect → Habitual PA)

To test Hypothesis B, we calculated the effects of the four main predictors of affective response (FS pre, FS midpoint, FS post, FS post 10) on habitual PA (LPA Move, MVPA Move, LPA Fitbit, MVPA Fitbit) while controlling for sociodemographic predictors (sex, age, BMI), resulting in 16 different models (see Table 6). Based on the *F*‐tests, all models showed significant associations, thus indicating that a model as a whole contributes to the explanation, with a range from 4.3% (FS midpoint—Fitbit LPA) to 12.6% (FS midpoint—Fitbit MVPA). In Model 1a, affect before the exercise test and age emerged as significant predictors, indicating that individuals who reported more positive affect prior to the exercise test, as well as older participants, had higher levels of habitual LPA. In Model 3a (affect before the exercise test predicting Fitbit‐derived LPA), affect itself was not a significant predictor in this model, but both sex and age showed significant positive associations, indicating that women and older individuals accumulated more habitual LPA. In Model 4a (affect before the exercise test predicting Fitbit‐derived MVPA), affect before and sex significantly predicted MVPA, indicating that participants who reported more positive affect prior to the exercise test and male participants engaged in more habitual MVPA. In Model 4b (midpoint affect during the exercise test predicting Fitbit‐derived MVPA), affect was a significant positive predictor and sex was a negative predictor, indicating that participants who experienced more positive affect at the midpoint of the exercise test, as well as male participants, engaged in higher levels of habitual MVPA as measured by Fitbit. In Model 3b (midpoint affect during the exercise test, predicting Fitbit‐derived LPA), only sex and age were significantly associated with LPA. In Models 3c and d (affect immediately and 10 min after the exercise test, predicting Fitbit‐derived LPA), only sex and age emerged as significant predictors, indicating that women and older participants engaged in more habitual LPA. In contrast, Models 4c and d (affect immediately and 10 min after the exercise test, predicting Fitbit‐derived MVPA) indicate that females engaged in less habitual MVPA.

**TABLE 6:** Multiple regression models for Hypothesis B.

	Model 1 (LPA Move)	Model 2 (MVPA Move)	Model 3 (LPA Fitbit)	Model 4 (MVPA Fitbit)
(a) FS pre	5.670 (1.983)*	0.007 (0.612)	3.701 (2.883)	3.749 (1.407)*
Sex	3.161 (5.855)	1.995 (1.807)	21.770 (8.405)*	−13.709 (4.101)*
Age	0.740 (0.277)*	−0.026 (0.086)	0.946 (0.405)*	−0.297 (0.197)
BMI	−0.782 (0.883)	0.062 (0.273)	−1.088 (1.249)	0.483 (0.609)
(b) FS midpoint	3.417 (2.019)	0.622 (0.613)	1.414 (2.870)	4.562 (1.378)*
Sex	2.561 (5.964)	2.297 (1.810)	21.275 (8.464)*	−13.139 (4.065)*
Age	0.761 (0.288)	−0.004 (0.087)	0.960 (0.417)*	−0.178 (0.200)
BMI	−0.384 (0.898)	0.094 (0.273)	−0.850 (1.256)	0.883 (0.603)
(c) FS post	0.614 (1.258)	−0.008 (0.380)	2.788 (1.761)	0.581 (0.880)
Sex	0.776 (5.986)	1.934 (1.809)	20.339 (8.424)*	−14.981 (4.211)**
Age	0.643 (0.284)	−0.024 (0.086)	0.937 (0.406)*	−0.350 (0.203)
BMI	−0.579 (0.904)	0.058 (0.273)	−1.006 (1.245)	0.617 (0.622)
(d) FS post 10	1.818 (1.725)	0.016 (0.522)	2.955 (2.435)	2.254 (1.200)
Sex	1.142 (5.914)	1.994 (1.791)	21.253 (8.383)*	−14.348 (4.132)**
Age	0.682 (283)	−0.026 (0.086)	0.951 (0.405)*	−0.301 (0.200)
BMI	−0.581 (0.896)	0.062 (0.272)	−0.938 (1.243)	0.639 (0.612)

*Note: B* (SE), unstandardized estimates and standard errors.

* *p* < 0.05.

** *p* < 0.001.

### 3.4 Hypothesis C (Executive Functions → Habitual PA)

To test Hypothesis B, we calculated the effects of the three main predictors of executive functions (absolute score (numerical updating task), switch cost (task switching), difference of reaction time congruent and incongruent (Stroop)) on habitual PA (LPA Move, MVPA Move, LPA Fitbit, MVPA Fitbit) while controlling for sociodemographic predictors (sex, age, BMI), thus resulting in 12 different models (see Table 7). Based on the *F*‐tests, all models showed significant associations, thus indicating that a model as a whole contributes to the explanation, with a range from 4.1% (updating task—Fitbit LPA) to 11.3% (switch cost—Fitbit MVPA). Overall, only in Model 4b does the main predictor, switch cost, become a significant predictor of Fitbit‐derived MVPA, indicating that better task‐switching performance was associated with higher habitual levels of MVPA. In line with the previously reported results, the covariate sex was a significant predictor in both Fitbit models, indicating that male participants engaged in more habitual MVPA and females in more habitual LPA. Further, age was a significant predictor in Models 3a, 3b, and 3c, indicating that older participants spent more time in habitual LPA.

**TABLE 7:** Multiple regression models for Hypothesis C.

	Model 1 (LPA Move)	Model 2 (MVPA Move)	Model 3 (LPA Fitbit)	Model 4 (MVPA Fitbit)
(a) Working memory (numerical updating task)	0.115 (0.252)	−0.043 (0.076)	0.137 (0.376)	−0.053 (0.184)
Sex	1.281 (5.866)	1.778 (1.771)	20.293 (8.383)*	−14.905 (4.112)**
Age	0.709 (0.288)	−0.050 (0.087)	0.947 (0.416)*	−0.353 (0.204)
BMI	−0.578 (0.897)	0.065 (0.271)	−0.893 (1.258)	0.645 (0.617)
(b) Cognitive flexibility (task switching)	−0.016 (0.010)	−0.005 (0.003)	−0.017 (0.014)	−0.019 (0.007)*
Sex	1.307 (5.820)	1.916 (1.757)	20.542 (8.339)*	−14.258 (4.011)**
Age	0.749 (0.280)	−0.014 (0.085)	0.986 (0.407)*	−0.249 (−0.196)
BMI	−0.521 (0.889)	0.115 (0.268)	−0.864 (1.245)	0.771 (−599)
(c) Inhibition control (Stroop)	0.008 (0.031)	−0.003 (0.009)	−0.041 (0.045)	0.018 (0.022)
Sex	0.972 (5.871)	1.889 (1.773)	20.834 (8.383)*	−15.161 (4.113)**
Age	0.655 (0.288)	−0.030 (0.087)	1.036 (0.425)*	−0.395 (0.209)
BMI	−0.608 (0.894)	0.077 (0.270)	−1.025 (1.248)	0.700 (0.612)

*Note: B* (SE), unstandardized estimates and standard errors.

* *p* < 0.05.

** *p* < 0.001.

### 3.5 Exploratory Analyses

We extended our analyses in an exploratory manner, thereby testing potential mediation effects of Path C. In particular, we tested, for each association between executive functions and habitual PA paramters, whether affective responses (pre, midpoint, post, and 10 min post) may mediate the effects. Overall, none of the conducted mediation analyses have shown significant indirect effects; therefore, they do not support the assumption of potential mediation effects (see Supporting Information S1: Table S3).

Furthermore, we incorporated sleep and sedentary behavior into the executive function and affect framework. Significant regressions were observed for several models across Paths B and C (see Supporting Information S1: Tables S1 and S2). In particular, significant associations were observed between affective states and sleep duration measured via both Move and Fitbit data. Model 1a revealed that FS pre was negatively associated with sleep duration measured with Move, indicating that higher affect before the exercise test was associated with shorter habitual sleep durations. According to Path C, switch cost was a significant predictor of sleep duration measured by Move, indicating that higher switch costs were associated with longer habitual sleep durations.

## 4 Discussion

This study aimed to evaluate the pathways of the neurocognitive‐affective model in a sample of healthy working adults. Overall, our analyses indicate significant associations for some model paths. In particular, affective states before and PA‐induced affective responses during exercise were significantly associated with habitual MVPA, as well as with habitual LPA minutes. Notably, the results differed in terms of the wearable device used. Further, our data indicate that cognitive flexibility was associated with habitual MVPA minutes measured by Fitbit, whereas working memory and inhibition control did not correlate with habitual PA. However, contrary to our expectations, no association was observed between cognitive flexibility, working memory, or inhibition control and PA‐induced affective response parameters. Exploratory analyses have shown that the extension of the neurocognitive‐affective model to the 24‐h movement behavior approach appears reasonable in terms of sleep. Furthermore, we identified no mediation effect of affective responses on the association between executive functions and habitual PA.

Path A shows no significant associations. In line with an earlier study (Brose et al. 2014), this study did not find a relationship between working memory performance and affective response. In contrast to the study by Khan and Siddiqui, which identified a significant relationship between higher cognitive flexibility and greater life satisfaction, this study demonstrates no association between higher cognitive flexibility and improved well‐being (Khan and Siddiqui 2023). In this context, well‐being can be seen as a determinant of health, representing a factor that determines life satisfaction (Priem and Schupp 2014). Moreover, in contrast to our findings, Wang et al. (2024) reported that cognitive flexibility was linked to reduced negative affect. A potential explanation for the discrepancy between the current findings and previous literature is that the sample may have exhibited ceiling effects across all three cognitive assessments, resulting in reduced variance between participants. Future research should prioritize increasing sample heterogeneity by incorporating participants from a broader range of occupational groups.

This study demonstrates associations in Path B of affective response before and PA induced during exercise on increased minutes in habitual MVPA measured by Fitbit, as well as between affective response before activity and increased minutes in habitual LPA measured by Move. Accordingly, previous research on the relationship between affect and PA has identified associations between higher positive affect and higher activity levels (Difrancesco et al. 2022; Li et al. 2022; Liao et al. 2017; Timm et al. 2024). The findings of previous studies (Le et al. 2021; Ruissen et al. 2022), which established a link between increased minutes in MVPA and higher positive affect, further support the results of this study. Notably, our findings varied depending on the wearable device used. Specifically, we observed stronger associations between affective states and the Fitbit than with the Move device. The significant differences between the Fitbit and Move data may be explained by differences in activity level classification. Although raw data from the Move sensor are available, no validated thresholds for classifying activity levels currently exist. In contrast, Fitbit leverages extensive user data and likely employs proprietary algorithms for threshold classification, though the details of these algorithms are not disclosed. Another potential explanation for the discrepancies is the difference in wearing positions. The Fitbit, worn on the wrist, may capture more movement during daily activities (e.g., cutting vegetables), potentially leading to an overestimation of PA (Feehan et al. 2018). This is reflected in the marked difference in daily MVPA minutes, with the Fitbit reporting nearly three times more than the Move sensor, which is worn on the thigh. This highlights the importance of careful consideration when planning a research study and selecting an appropriate wearable for measuring MVPA.

Path C indicates that better task‐switching performance is associated with a higher number of minutes spent in MVPA. In contrast, no significant associations were found between PA parameters and working memory or inhibitory control performance. Consistent with our findings, a study by Martínez‐López et al. (2021) demonstrated a positive association between MVPA and cognitive flexibility in school‐aged children. Further, Angevaren et al. (2007) showed that the intensity of weekly physical activity was significantly and positively related to cognitive flexibility. Although Gothe reported a general positive relationship between executive functions and PA (Gothe 2021), Kato et al. identified significant positive associations specifically between working memory and PA (Kato et al. 2018). Similarly, Salas‐Gomez et al. found positive associations between inhibitory control and PA (Salas‐Gomez et al. 2020). These associations, however, were not supported in this study. The results for these two pathways are, therefore, inconclusive at the between‐subjects level, which was the focus of this analysis. Future research should explore potential associations at the within‐subject level, which could be investigated using ambulatory assessment methods (Reichert et al. 2020). Additionally, the model could be evaluated at different temporal resolutions (e.g., momentary or daily associations) to uncover potential effects. This, however, presents the challenge that cognitive tests in everyday settings need to be adaptive or exhibit sufficient variability to account for learning effects.

Expanding the focus from a single‐behavior perspective to a multibehavior approach aligns with the growing recognition of the 24‐h movement behavior paradigm in PA research. This paradigm encompasses all daily movement and nonmovement behaviors, including sleep, PA, and sedentary behavior (Stevens et al. 2020). Recent studies utilizing compositional data analysis have highlighted its value in elucidating associations between physical behavior compositions and health outcomes (Blodgett et al. 2023), offering insights into the optimal balance of time spent in PA, sedentary behavior, and sleep for health improvements. Our exploratory analyses demonstrated significant associations for Paths B and C, not only with PA but also with sleep variables. For instance, higher affective responses to PA before activity were significantly related to reduced sleep, as recorded by the Move. Additionally, better task‐switching performance was associated with longer sleep duration, and longer sleep duration was associated with better task‐switching performance, as measured by the Move device.

Some limitations merit further discussion. First, the cognitive tests were administered at varying times of the day, with some participants completing them in the morning before work and others in the afternoon after their workday. This variability in timing may have influenced cognitive performance. Second, each of the three executive functions was measured with only one task. As Miyake et al. (2000) point out, single tasks often capture substantial nonexecutive and common executive variance, limiting construct validity. We emphasize that future studies should use multiple tasks and latent factor scores for a more precise assessment. Third, the Move device assessed sedentary behavior based solely on body posture, whereas current literature emphasizes that energy expenditure should also be considered alongside posture. Additionally, the Move is neither shockproof nor waterproof, preventing its use during activities such as swimming or contact sports, which may have led to incomplete activity data. Fourth, the study sample consisted exclusively of healthy university employees, limiting its representativeness and, consequently, the generalizability of the findings to broader populations. Therefore, further research is needed to address these limitations. Further, because weekend days were deliberately excluded as break days, this may have led to an underestimation of leisure‐time PA in the assessment. Last, the absence of a sleep log to verify or complement the device‐based sleep detection represents a limitation. Without subjective reports on sleep onset and wake times, potential inaccuracies in the algorithm‐based sleep estimates cannot be ruled out. Additionally, no specific inclusion criteria were applied exclusively to the sleep data (e.g., minimum number of valid nights or hours of sleep), which may affect the consistency and comparability of the sleep‐related results. Despite these limitations, it is worth noting that the sample size of this study is approximately seven times larger than that of the first model evaluation (Loprinzi et al. 2020).

## 5 Conclusion

This study is the second to evaluate the neurocognitive‐affective model and provides partial empirical support for its pathways in healthy working adults. PA‐induced affective responses were significantly associated with habitual MVPA and LPA, particularly when measured with a commercial device (Fitbit), underscoring the importance of device selection in research. Cognitive flexibility was linked to habitual MVPA (Path C), but not to affective responses, whereas no significant associations emerged for working memory or inhibitory control. These findings advance our understanding of the interplay among PA, cognition, and affect and highlight the value of theory‐driven study designs. To strengthen future research in this field, theoretical models should be more consistently used as a conceptual foundation for study design, as they promote conceptual clarity, guide hypothesis development, and support cumulative scientific progress. By identifying the roles of cognitive flexibility and affect in promoting PA, our results can inform targeted interventions to enhance PA engagement and well‐being. Moreover, extending the neurocognitive‐affective model to include 24‐h movement behaviors such as sleep offers a broader perspective on health‐related outcomes, supporting more effective public health strategies and personalized behavior change programs.

## Conflicts of Interest

The authors declare no conflicts of interest.

## Supporting information


Supporting Information S1

## Data Availability

The authors confirm that the data supporting the findings of this study are available on request.
