# Supplementary material for: Evaluation of the Neurocognitive Affective Model for the Prediction of Habitual 24‐Hour Physical Behavior
Source: Eur J Sport Sci. 2025 Aug 12;25(9):e70037. doi: 10.1002/ejsc.70037 (PMC12342056; doi:10.1002/ejsc.70037)
Supplement: Supplementary file 1 — Supporting Information S1 [file EJSC-25-e70037-s001.docx]

**Supporting information**

Table S1. Multiple regression models for exploratory analyses of hypothesis B

|  | **Model 1**  **(Sleep Move)** | **Model 2**  **(Sedentary Move)** | **Model 3**  **(Sleep Fitbit)** | **Model 4**  **(Sedentary Fitbit)** |
| --- | --- | --- | --- | --- |
| **a) FS pre** | -7.778(2.781) * | -1.018(3.482) | -4.279(2.470) | -6.898(3.291) |
| Sex | -14.611(8.211) | 8.884(10.282) | 3.634(7.201) | -12.559(9.592) |
| Age | -1.662(.389) ** | .593(.487) | -1.011(.347) * | .443(.462) |
| BMI | 1.096(1.239) | .469(1.551) | -.111(1.070) | 1.622(1.425) |
| **b) FS midpoint** | -2.460(2.845) | -2.913(3.492) | -3.103(2.459) | -5.899(3.273) |
| Sex | -12.698(8.404) | 7.867(10.313) | 3.704(7.251) | -12.753(9.651) |
| Age | -1.614(.406) ** | .508(.498) | -1.076(.357) * | .307(.475) |
| BMI | .665(1.265) | .279(1.553) | -.461(1.076) | 1.014(1.432) |
| **c) FS post** | .588(1.762) | -2.667(2.154) | -.713(1.530) | -1.995(2.039) |
| Sex | -11.353(8.381) | 8.802(10.247) | 4.571(7.320) | -9.720(9.756) |
| Age | -1.505(.397) ** | .595(.485) | -.950(.352) | .490(.470) |
| BMI | .798(1.266) | .433(1.548) | -.301(1.081) | 1.424(1.441) |
| **d) FS post 10** | -2.735(2.416) | -.622(2.974) | -2.016(2.098) | -2.857(2.806) |
| Sex | -11.874(8.283) | 9.206(10.197) | 4.451(7.225) | -11.180(9.661) |
| Age | -1.589(.396) ** | .597(.488) | -.998(349) | .468(.467) |
| BMI | .822(1.255) | .437(1.546) | -.293(1.071) | 1.328(1.432) |
| Note: B(SE), Unstandardized estimates and standard errors; * p<.05; ** p<.001 | | | | |

Table S2. Multiple regression models for exploratory analyses of hypothesis C

|  | **Model 1**  **(Sleep Move)** | **Model 2**  **(Sedentary Move)** | **Model 3**  **(Sleep Fitbit)** | **Model 4**  **(Sedentary Fitbit)** |
| --- | --- | --- | --- | --- |
| **a) Working Memory**  **(Numerical Updating Task)** | .306(.356) | -.524(.436) | .216(.295) | -.226(.416) |
| Sex | -10.446(8.280) | 6.637(10.151) | 7.703(6.805) | -8.432(9.591) |
| Age | -1.501(.407) ** | .463(.498) | -.959(.333) * | .546(.469) |
| BMI | 1.001(1.267) | .277(1.553) | -.467(1.033) | 1.570(1.456) |
| **b) Cognitive Flexibility**  **(Task Switching)** | .052(.014) ** | -.027(.018) | .018(.012) | .019(.017) |
| Sex | -11.678(7.992) | 7.880(10.107) | 6.784(6.775) | -8.879(9.569) |
| Age | -1.840(.385) ** | .748(.487) | -1.094(.320) ** | .551(.452) |
| BMI | .531(1.221) | .667(1.544) | -.678(1.024) | 1.529(1.446) |
| **c) Inhibition Control**  **(Stroop)** | .036(.044) | -.030(.054) | .060(.037) | -.007(.052) |
| Sex | -11.480(8.285) | 7.939(10.185) | 6.275(6.792) | -8.030(9.639) |
| Age | -1.682(.406) ** | .694(.500) | -1.203(.334) ** | .640(.474) |
| BMI | .952(1.262) | .428(1.551) | -.460(1.021) | 1.651(1.450) |
| Note: B(SE), Unstandardized estimates and standard errors; * p<.05; ** p<.001 | | | | |

Table S3: Meditation analyses between executive functions, affective responses, and habitual PA parameters.

| **X** | **M** | **Y** | **Boot LLCI** | **Boot ULCI** |
| --- | --- | --- | --- | --- |
| Switch_c | FS_pre | MVPA_FITBIT | -0.0028 | 0.0023 |
| Switch_c | FS_midpo | MVPA_FITBIT | -0.0059 | 0.0022 |
| Switch_c | FS_post | MVPA_FITBIT | -0.0057 | 0.0011 |
| Switch_c | FS_post_10 | MVPA_FITBIT | -0.0021 | 0.0023 |
| Switch_c | FS_pre | LPA_FITBIT | -0.0046 | 0.0034 |
| Switch_c | FS_midpo | LPA_FITBIT | -0.0034 | 0.0090 |
| Switch_c | FS_post | LPA_FITBIT | -0.0004 | 0.0172 |
| Switch_c | FS_post_10 | LPA_FITBIT | -0.0051 | 0.0049 |
| Switch_c | FS_pre | MVPA_MOVE | -0.0006 | 0.0007 |
| Switch_c | FS_midpo | MVPA_MOVE | -0.0014 | 0.0006 |
| Switch_c | FS_post | MVPA_MOVE | -0.0015 | 0.0014 |
| Switch_c | FS_post_10 | MVPA_MOVE | -0.0008 | 0.0010 |
| Switch_c | FS_pre | LPA_MOVE | -0.0053 | 0.0036 |
| Switch_c | FS_midpo | LPA_MOVE | -0.0022 | 0.0030 |
| Switch_c | FS_post | LPA_MOVE | -0.0039 | 0.0057 |
| Switch_c | FS_post_10 | LPA_MOVE | -0.0027 | 0.0041 |
| WM_score | FS_pre | MVPA_FITBIT | -0.0177 | 0.1551 |
| WM_score | FS_midpo | MVPA_FITBIT | -0.0252 | 0.2510 |
| WM_score | FS_post | MVPA_FITBIT | -0.0373 | 0.1294 |
| WM_score | FS_post_10 | MVPA_FITBIT | -0.0403 | 0.0393 |
| WM_score | FS_pre | LPA_FITBIT | -0.0979 | 0.2925 |
| WM_score | FS_midpo | LPA_FITBIT | -0.3452 | 0.0426 |
| WM_score | FS_post | LPA_FITBIT | -0.2365 | 0.0817 |
| WM_score | FS_post_10 | LPA_FITBIT | -0.0718 | 0.1030 |
| WM_score | FS_pre | MVPA_MOVE | -0.0311 | 0.0274 |
| WM_score | FS_midpo | MVPA_MOVE | -0.0140 | 0.0438 |
| WM_score | FS_post | MVPA_MOVE | -0.0129 | 0.0318 |
| WM_score | FS_post_10 | MVPA_MOVE | -0.0179 | 0.0185 |
| WM_score | FS_pre | LPA_MOVE | -0.0081 | 0.2800 |
| WM_score | FS_midpo | LPA_MOVE | -0.0775 | 0.0878 |
| WM_score | FS_post | LPA_MOVE | -0.0632 | 0.0709 |
| WM_score | FS_post_10 | LPA_MOVE | -0.0963 | 0.0489 |
| RTdiff_inkon_kon | FS_pre | MVPA_FITBIT | -0.0146 | 0.0048 |
| RTdiff_inkon_kon | FS_midpo | MVPA_FITBIT | -0.0321 | 0.0079 |
| RTdiff_inkon_kon | FS_post | MVPA_FITBIT | -0.0102 | 0.0097 |
| RTdiff_inkon_kon | FS_post_10 | MVPA_FITBIT | -0.0090 | 0.0056 |
| RTdiff_inkon_kon | FS_pre | LPA_FITBIT | -0.0260 | 0.0084 |
| RTdiff_inkon_kon | FS_midpo | LPA_FITBIT | -0.0103 | 0.0428 |
| RTdiff_inkon_kon | FS_post | LPA_FITBIT | -0.0210 | 0.0216 |
| RTdiff_inkon_kon | FS_post_10 | LPA_FITBIT | -0.0191 | 0.0127 |
| RTdiff_inkon_kon | FS_pre | MVPA_MOVE | -0.0024 | 0.0026 |
| RTdiff_inkon_kon | FS_midpo | MVPA_MOVE | -0.0060 | 0.0026 |
| RTdiff_inkon_kon | FS_post | MVPA_MOVE | -0.0037 | 0.0016 |
| RTdiff_inkon_kon | FS_post_10 | MVPA_MOVE | -0.0025 | 0.0030 |
| RTdiff_inkon_kon | FS_pre | LPA_MOVE | -0.0233 | 0.0096 |
| RTdiff_inkon_kon | FS_midpo | LPA_MOVE | -0.0111 | 0.0096 |
| RTdiff_inkon_kon | FS_post | LPA_MOVE | -0.0086 | 0.0075 |
| RTdiff_inkon_kon | FS_post_10 | LPA_MOVE | -0.0082 | 0.0116 |
| RTdiff_inkon_kon | FS_pre | Sleep_FITBIT | -0.0087 | 0.0244 |
| RTdiff_inkon_kon | FS_midpo | Sleep_FITBIT | -0.0238 | 0.0125 |
| RTdiff_inkon_kon | FS_post | Sleep_FITBIT | -0.0097 | 0.0088 |
| RTdiff_inkon_kon | FS_post_10 | Sleep_FITBIT | -0.0152 | 0.0146 |
| RTdiff_inkon_kon | FS_pre | Sedentary_FITBIT | -0.0093 | 0.0335 |
| RTdiff_inkon_kon | FS_midpo | Sedentary_FITBIT | -0.0111 | 0.0455 |
| RTdiff_inkon_kon | FS_post | Sedentary_FITBIT | -0.0118 | 0.0105 |
| RTdiff_inkon_kon | FS_post_10 | Sedentary_FITBIT | -0.0225 | 0.0166 |
| RTdiff_inkon_kon | FS_pre | Sleep_MOVE | -0.0160 | 0.0306 |
| RTdiff_inkon_kon | FS_midpo | Sleep_MOVE | -0.0273 | 0.0123 |
| RTdiff_inkon_kon | FS_post | Sleep_MOVE | -0.0106 | 0.0120 |
| RTdiff_inkon_kon | FS_post_10 | Sleep_MOVE | -0.0104 | 0.0160 |
| RTdiff_inkon_kon | FS_pre | Sedentary_MOVE | -0.0114 | 0.0179 |
| RTdiff_inkon_kon | FS_midpo | Sedentary_MOVE | -0.0128 | 0.0307 |
| RTdiff_inkon_kon | FS_post | Sedentary_MOVE | -0.0173 | 0.0119 |
| RTdiff_inkon_kon | FS_post_10 | Sedentary_MOVE | -0.0244 | 0.0122 |
